# Supplementary material for: Topological data analysis for predicting disease outbreaks in humanitarian settings: A machine learning approach
Source: PLoS One. 2026 Jun 5;21(6):e0350644. doi: 10.1371/journal.pone.0350644 (PMC13240865; doi:10.1371/journal.pone.0350644)
Supplement: S1 Appendix — Complete list of the 15 raw variables and 25 topological features used in the final model, including variable descriptions, data sources, and data processing notes. See also S1 Table. (PDF) [file pone.0350644.s001.pdf]

S1 Appendix. Data Dictionary.

This appendix provides a complete data dictionary for all variables used in the final XGBoost-TDA model. The model includes 15 raw features and 25 topological features derived from persistent homology computation.

Raw Features (n=15)

Climate Variables (n=2)

Precipitation anomaly: Deviation from the historical mean precipitation for the LGA and epidemiological week, computed from CHIRPS data. Positive values indicate above-average rainfall.

Temperature anomaly: Deviation from historical mean temperature from ERA5 reanalysis data.

Conflict Variables (n=2)

Conflict events: Weekly count of violent events (battles, violence against civilians, explosions/remote violence, riots, and protests) from the Armed Conflict Location & Event Data Project (ACLED).

Conflict fatalities: Weekly fatalities resulting from violent conflict events from ACLED.

Displacement Variables (n=1)

IDP concentration: Number of internally displaced persons per 100,000 population, from the International Organization for Migration (IOM) Displacement Tracking Matrix (DTM).

Health System Variables (n=2)

Vaccination coverage: Routine immunization coverage (%) estimated by WHO/UNICEF.

Health facility density: Number of health facilities per 100,000 population from the Nigeria Health Facility Registry.

Socioeconomic Variables (n=9)

Poverty rate: Population below the poverty line (%) from the Nigeria Living Standards Measurement Study (LSMS).

Water access: Percentage of population with access to improved water sources from Demographic and Health Surveys (DHS).

Sanitation access: Percentage of population with access to improved sanitation from DHS.

Population density: Persons per km2 from WorldPop.

Weeks since last outbreak: Time (in weeks) since the previous outbreak in the same LGA, computed separately for cholera and measles from NCDC surveillance data.

Drought index: Palmer Drought Severity Index from NOAA.

Political stability: Political stability index from Worldwide Governance Indicators (WGI).

Market price index: Food price index from the World Food Programme (WFP).

Topological Features (n=25)

All topological features were computed using GUDHI 3.8.0 with Vietoris-Rips filtrations across 100 logarithmically-spaced scale parameters (epsilon = 0.01 to 10.0).

beta_0 mean: Mean number of connected components (0-dimensional homology) across the filtration. Interpreted as a fragmentation index.

beta_0 max: Maximum number of connected components observed during the filtration.

beta_0 SD: Standard deviation of connected component counts across the filtration.

beta_1 mean: Mean number of 1-dimensional loops (1-dimensional homology) across the filtration. Interpreted as a cyclic dependency index.

beta_1 max: Maximum number of 1-dimensional loops observed during the filtration.

beta_1 SD: Standard deviation of 1-dimensional loop counts across the filtration.

Persistence entropy: Entropy of the persistence distribution, computed as H = -sum(p_i log(p_i)) where p_i = persistence_i / sum(persistence_j).

Total persistence: Sum of all persistence values across all topological features.

Persistence landscape 1-17: Vectorized persistence landscape values (17 components from the discretized landscape function).

Data Processing Notes

All 57 input features were standardized (z-score normalization) before computing Euclidean distances for the Vietoris-Rips filtration. Missing values (3-5% across variables) were imputed using Multiple Imputation by Chained Equations (MICE) with 10 imputations.

See also S1 Table for a summary of all variables and their sources.
