# Supplementary material for: Topological data analysis for predicting disease outbreaks in humanitarian settings: A machine learning approach
Source: PLoS One. 2026 Jun 5;21(6):e0350644. doi: 10.1371/journal.pone.0350644 (PMC13240865; doi:10.1371/journal.pone.0350644)
Supplement: S2 Appendix — Optimized XGBoost hyperparameters for both XGBoost-Raw and XGBoost-TDA models, including learning rate, max depth, subsample ratio, and regularization parameters. Details on the Bayesian optimization procedure and training configuration. Hyperparameters were optimized via Bayesian optimization with 50 iterations using the same search space for both models. See also S2 Table. (PDF) [file pone.0350644.s002.pdf]

S2 Appendix. Model Hyperparameters.

This appendix provides the optimized XGBoost hyperparameters for both XGBoost-Raw and XGBoost-TDA models. Hyperparameters were optimized via Bayesian optimization with 50 iterations within each cross-validation training fold.

Optimization Method

Bayesian optimization was performed using scikit-optimize with 50 iterations per fold. The same search space was used for both XGBoost-Raw (15 raw features only) and XGBoost-TDA (15 raw + 25 topological features) to ensure fair comparison. The search space included: learning rate (0.01-0.3), max depth (3-10), subsample ratio (0.5-1.0), column sample ratio (0.5-1.0), L2 regularization (0.1-10.0), min child weight (1-10), and number of estimators (100-1000).

Optimized Hyperparameters

XGBoost-Raw (15 features)

Learning rate (eta): 0.05

Max depth: 6

Subsample ratio: 0.80

Column sample ratio: 0.80

L2 regularization (lambda): 1.0

Min child weight: 3

Number of estimators: 500

Early stopping rounds: 50

XGBoost-TDA (40 features)

Learning rate (eta): 0.04

Max depth: 7

Subsample ratio: 0.75

Column sample ratio: 0.75

L2 regularization (lambda): 1.5

Min child weight: 3

Number of estimators: 600

Early stopping rounds: 50

Other Fixed Parameters

Objective: binary:logistic

Evaluation metric: logloss

Scale pos weight: class_weight="balanced" (automatically adjusted inversely proportional to class frequencies)

Random seed: 42 (for reproducibility)

Training Details

Nested cross-validation was used: the outer loop had 5 temporal folds (train on 2018 through 2018+k, test on 2019+k, for k=0,...,4), and the inner loop used 3-fold cross-validation on the training data only for hyperparameter tuning. The final hold-out set (last 6 months of 2023) was not used during model development or hyperparameter selection.

See also S2 Table for a summary of optimized hyperparameters.
