# Supplementary material for: Topological data analysis for predicting disease outbreaks in humanitarian settings: A machine learning approach
Source: PLoS One. 2026 Jun 5;21(6):e0350644. doi: 10.1371/journal.pone.0350644 (PMC13240865; doi:10.1371/journal.pone.0350644)
Supplement: S3 Appendix — Supplementary performance visualizations including: (a) Precision-recall curves for cholera and measles prediction models; (b) Calibration curves for XGBoost-TDA model; (c) Software environment details and computational resources. See also S1 Fig and S2 Fig. (PDF) [file pone.0350644.s003.pdf]

S3 Appendix. Additional Performance Metrics.

This appendix provides supplementary performance visualizations and additional metrics not included in the main manuscript.

(a) Precision-Recall Curves

Precision-recall curves for all models (cholera and measles) are provided in S1 Fig. XGBoost-TDA achieves the highest precision-recall AUC for both diseases, with particularly strong performance for cholera prediction in the high-recall operating region relevant for early warning applications.

Cholera Precision-Recall AUC Results:

XGBoost-TDA: 0.36 (95% CI: 0.32-0.40)

XGBoost-Raw: 0.31 (95% CI: 0.27-0.35)

Random Forest: 0.28 (95% CI: 0.24-0.32)

Logistic Regression: 0.22 (95% CI: 0.19-0.26)

Measles Precision-Recall AUC Results:

XGBoost-TDA: 0.32 (95% CI: 0.28-0.36)

XGBoost-Raw: 0.27 (95% CI: 0.23-0.31)

Random Forest: 0.24 (95% CI: 0.20-0.28)

Logistic Regression: 0.18 (95% CI: 0.15-0.22)

(b) Calibration Curves

Calibration curves for the XGBoost-TDA model are provided in S2 Fig. The calibration plots show observed frequency versus predicted probability for both cholera and measles. The model demonstrates acceptable calibration with slopes close to 1.0 (0.94 for cholera, 0.97 for measles), indicating that predicted probabilities accurately reflect true event likelihoods.

(c) Software Environment Details

All analyses were conducted using Python 3.9 with the following package versions:

scikit-learn: 1.3.0 (machine learning framework)

XGBoost: 2.0.0 (gradient boosting)

GUDHI: 3.8.0 (persistent homology computation)

SHAP: 0.42.0 (model interpretability)

SciPy: 1.11.0 (statistical tests)

pandas: 2.0.0 (data manipulation)

numpy: 1.24.0 (numerical computing)

scikit-optimize: 0.9.0 (Bayesian optimization)

matplotlib: 3.7.0 (visualization)

seaborn: 0.12.0 (statistical visualization)

Computational Resources

All computations were performed on a workstation with 32 GB RAM and an Intel Core i9 processor. Persistent homology computation for 25,284 observations with 57 features required approximately 4 hours using the GUDHI library with default parameters. Model training with Bayesian optimization required approximately 6 hours per fold.

See also S1 Fig and S2 Fig for the corresponding visualizations.
