# Supplementary material for: Topological data analysis for predicting disease outbreaks in humanitarian settings: A machine learning approach
Source: PLoS One. 2026 Jun 5;21(6):e0350644. doi: 10.1371/journal.pone.0350644 (PMC13240865; doi:10.1371/journal.pone.0350644)
Supplement: S1 Table — (PDF) [file pone.0350644.s004.pdf]

S1 Table. Complete list of the 15 raw variables and 25 topological features used in the final model.

Variable	Description	Source
Raw Features (n=15)		
Precipitation anomaly	Deviation from historical mean precipitation	CHIRPS
Temperature anomaly	Deviation from historical mean temperature	ERA5
Conflict events	Weekly count of violent events	ACLED
Conflict fatalities	Weekly fatalities from violent events	ACLED
IDP concentration	IDPs per 100,000 population	IOM DTM
Vaccination coverage	Routine immunization coverage (%)	WHO/UNICEF
Health facility density	Facilities per 100,000 population	Nigeria HFR
Poverty rate	Population below poverty line (%)	LSMS
Water access	Improved water source access (%)	DHS
Sanitation access	Improved sanitation access (%)	DHS
Population density	Persons per km2	WorldPop
Weeks since last outbreak	Time since previous outbreak in LGA	NCDC
Drought index	Palmer Drought Severity Index	NOAA
Political stability	Political stability index	WGI
Market price index	Food price index	WFP
Topological Features (n=25)		
beta_0 mean	Mean connected components across filtration	Computed
beta_0 max	Maximum connected components	Computed
beta_0 SD	Standard deviation of connected components	Computed
beta_1 mean	Mean 1-dimensional loops across filtration	Computed

Variable	Description	Source
beta_1 max	Maximum 1-dimensional loops	Computed
beta_1 SD	Standard deviation of 1-dimensional loops	Computed
Persistence entropy	Entropy of persistence distribution	Computed
Total persistence	Sum of all persistence values	Computed
Persistence landscape 1-17	Vectorized persistence landscape	Computed

CHIRPS = Climate Hazards Group InfraRed Precipitation with Station Data;

ERA5 = ECMWF Reanalysis v5; ACLED = Armed Conflict Location & Event Data Project;

IOM DTM = International Organization for Migration Displacement Tracking Matrix;

Nigeria HFR = Nigeria Health Facility Registry; LSMS = Living Standards Measurement Study;

DHS = Demographic and Health Surveys; WGI = Worldwide Governance Indicators; WFP = World Food Programme.
