# Supplementary material for: Topological data analysis for predicting disease outbreaks in humanitarian settings: A machine learning approach
Source: PLoS One. 2026 Jun 5;21(6):e0350644. doi: 10.1371/journal.pone.0350644 (PMC13240865; doi:10.1371/journal.pone.0350644)
Supplement: S2 Table — (PDF) [file pone.0350644.s005.pdf]

S2 Table. Optimized XGBoost hyperparameters for XGBoost-Raw and XGBoost-TDA models.

Parameter	XGBoost-Raw	XGBoost-TDA
Learning rate (eta)	0.05	0.04
Max depth	6	7
Subsample ratio	0.80	0.75
Column sample ratio	0.80	0.75
L2 regularization (lambda)	1.0	1.5
Min child weight	3	3
Number of estimators	500	600
Early stopping rounds	50	50

Hyperparameters optimized via Bayesian optimization with 50 iterations.

Same search space used for both models.

All other parameters used XGBoost defaults.
