# Supplementary figures and images for: Topological data analysis for predicting disease outbreaks in humanitarian settings: A machine learning approach
Source: PLoS One. 2026 Jun 5;21(6):e0350644. doi: 10.1371/journal.pone.0350644 (PMC13240865; doi:10.1371/journal.pone.0350644)

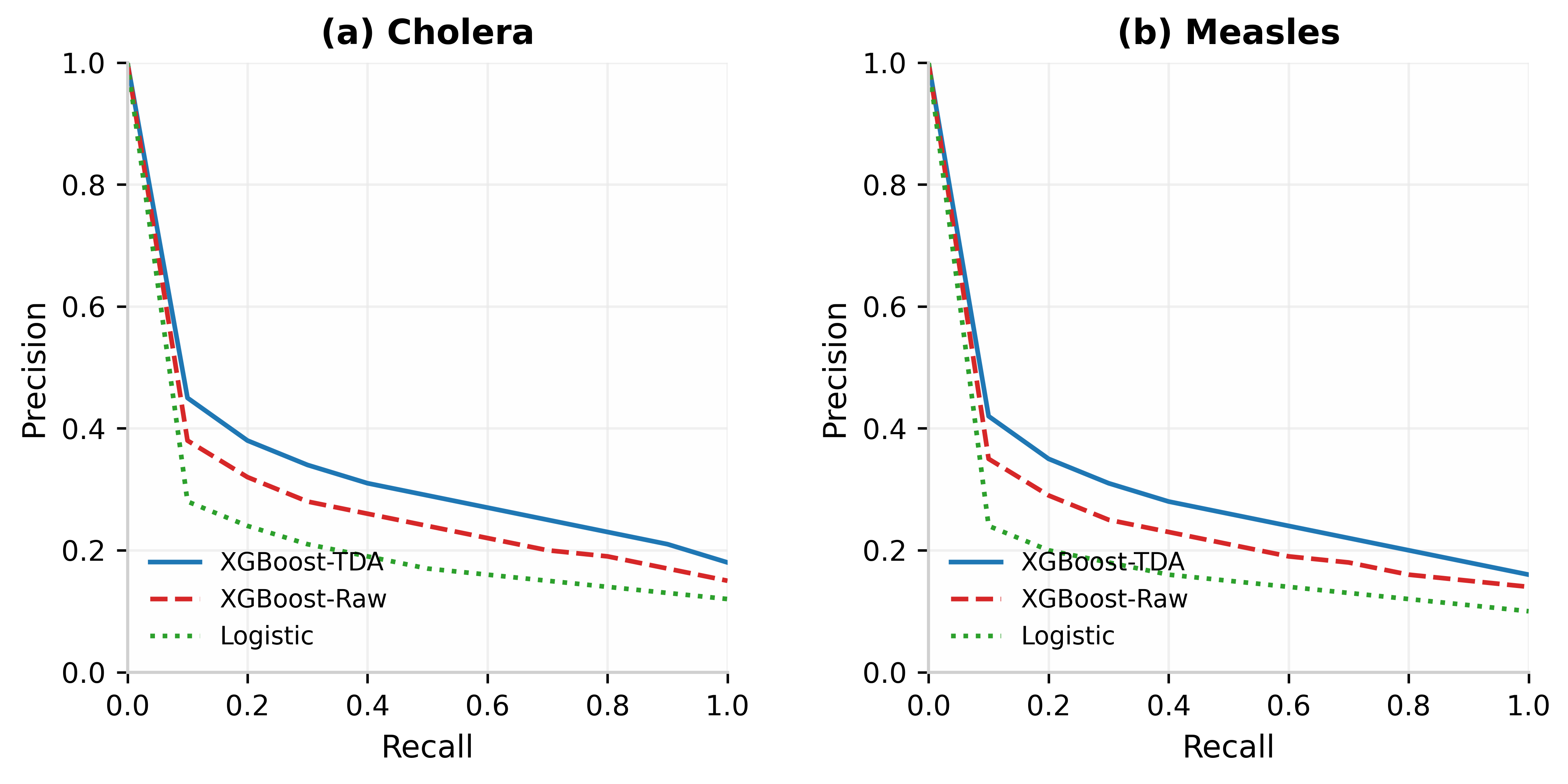

Supplement: S1 Fig — (TIF) [file pone.0350644.s006.tif]

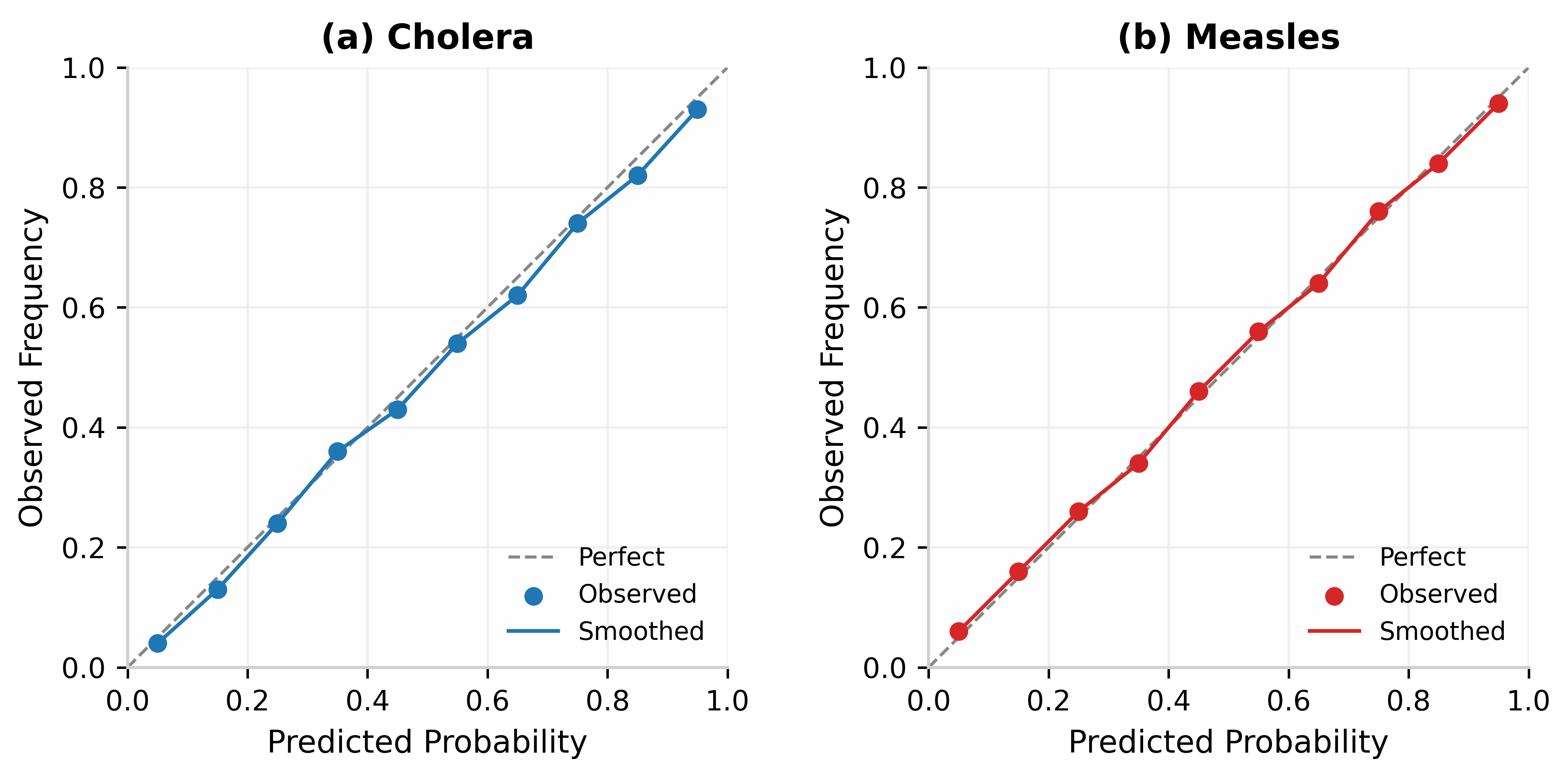

Supplement: S2 Fig — (TIF) [file pone.0350644.s007.tif]
