# Supplementary material for: Topological data analysis for predicting disease outbreaks in humanitarian settings: A machine learning approach
Source: PLoS One. 2026 Jun 5;21(6):e0350644. doi: 10.1371/journal.pone.0350644 (PMC13240865; doi:10.1371/journal.pone.0350644)
Supplement: Attachment — Submitted filename: Response_to_Reviewers.pdf [file pone.0350644.s011.pdf]

Response to Reviewers

Manuscript PONE-D-26-03649

Topological Data Analysis for Predicting Disease Outbreaks in Humanitarian Settings: A Machine Learning Approach

---

Authors: Job Agba Opue*, Uchechukwu Okorie
Corresponding Author: job.opue@covenantuniversity.edu.ng
Submission Date: April 2026

General Overview

We thank the Academic Editor and all ten reviewers for their thorough and constructive feedback on our initial submission. The reviewers identified critical issues regarding outcome definition, validation methodology, data availability, and manuscript structure that required substantial revision.

In response, we have undertaken a comprehensive revision that addresses all major concerns:

1. **Complete reframing of the research question:** We shifted from malnutrition surveillance to disease outbreak prediction (cholera and measles), where outcome definitions are clearer and validation more straightforward.

2. **Rigorous validation framework:** We implemented forward-chaining cross-validation with strict temporal ordering and a temporal hold-out set to prevent data leakage.

3. **Comprehensive outcome specification:** We provide explicit operational definitions using WHO surveillance thresholds, class distributions, and lead time calculations.

4. **Expanded analytical depth:** We added feature importance analysis, calibration assessment, ablation studies, subgroup analysis, and failure analysis.

5. **Data availability:** We provide complete data source information and commit to repository deposition.

6. **Restructured presentation:** The manuscript now follows standard scientific format with integrated abstract, proper literature review, and expanded results.

The revised manuscript represents a fundamentally improved contribution that addresses all reviewer concerns while maintaining the core innovation of applying topological data analysis

to humanitarian early warning.

Response to Academic Editor

Editor Comment 1: Outcome Variable Definition

The Outcome Variable is Not Adequately Defined. Multiple reviewers highlight this as a fatal flaw. The manuscript states that “elevated SAM risk” is defined either as a binary indicator or an ordinal category “depending on the operational outcome series available.” This is not acceptable. Questions that remain unanswered: What specific dataset provides the SAM outcome? What is the temporal resolution? What is the time period covered? How is a “surge” operationally defined? What threshold separates “normal” from “crisis”? What is the class distribution? How is lead time calculated?

Response

We completely agree with this assessment. The original manuscript’s vague outcome definition was a critical weakness. In the revised manuscript, we have:

- **Changed the outcome focus:** We now predict cholera and measles surge events using WHO surveillance thresholds, which have established operational definitions.
- **Explicit outcome definitions:**
  - Cholera surge: Alert threshold ($\geq$5 cases in a single week in an LGA with no previous cholera cases in current year) OR epidemic threshold (weekly case incidence $\geq$2 standard deviations above the 5-year historical mean)
  - Measles surge: Confirmed outbreak ($\geq$5 laboratory-confirmed cases in a single week) OR suspected outbreak ($\geq$10 suspected cases per 100,000 population with vaccination coverage $<$80%)
- **Complete outcome specification:** We report the class distribution (cholera: 7.0%, measles: 5.0%), temporal resolution (weekly), study period (2018–2023), total observations (25,284 LGA-weeks), and prediction horizon (4 weeks).
- **Lead time calculation:** The prediction target is surge occurrence in the subsequent 4-week window, providing operational lead time for preventive interventions.

Location in Revised Manuscript

Methods section, subsection “Outcome Definition” (Lines 184–195)
Results section, subsection “Descriptive Statistics” (Lines 284–310)

Editor Comment 2: Validation Framework

The Validation Framework is Inadequately Specified and Likely Optimistically Biased. Reviewers 2, 3, 4, 7, and 8 all raise serious concerns about potential temporal data leakage. The manuscript mentions “stratified K-fold cross-validation” but does not clarify whether folds are constructed in a time-aware manner. In a forecasting context with lagged predic-

tors and claimed lead times, standard random K-fold splitting is invalid.

Response

This was a critical methodological flaw in the original submission. We have completely redesigned the validation framework:

- **Forward-chaining cross-validation:** Five temporal folds respecting strict temporal ordering:
  - Fold 1: Training = 2018, Test = 2019
  - Fold 2: Training = 2018–2019, Test = 2020
  - Fold 3: Training = 2018–2020, Test = 2021
  - Fold 4: Training = 2018–2021, Test = 2022
  - Fold 5: Training = 2018–2022, Test = 2023
- **Nested cross-validation:** Hyperparameter tuning is performed within each training fold (inner 3-fold), ensuring no information leakage from test sets.
- **Temporal hold-out validation:** A final hold-out set (last 6 months of 2023) was reserved for unbiased performance estimation and never used during model development.
- **Performance with uncertainty:** We report ROC-AUC with 95% confidence intervals using bootstrap percentile method, and report fold-level variability (sensitivity range: 0.68–0.76 across folds).

Location in Revised Manuscript

Methods section, subsection "Validation Framework" (Lines 234–248)
Results section, subsection "Temporal Hold-Out Validation" (Lines 463–466)

Editor Comment 3: Spatial Coverage

The Spatial Coverage is Unclear and Potentially Misleading. Reviewer 2 identifies a critical discrepancy: the manuscript presents the framework as an LGA-level system for Nigeria, yet the pipeline figure specifies a point cloud of 97 LGAs. Nigeria has 774 LGAs. If only 97 were included, this represents a substantial spatial restriction that is neither clearly described nor justified.

Response

We acknowledge this discrepancy and have addressed it comprehensively:

- **Explicit spatial coverage:** We clearly state that 97 of 774 LGAs were included in the analysis (Lines 149–150).
- **Inclusion criteria:** These 97 LGAs represent high-burden areas with complete data across all domains and documented outbreak history, concentrated in the northeast

(Borno, Adamawa, Yobe) and northwest (Katsina, Zamfara, Sokoto) where conflict and displacement are most severe.

- **Representativeness analysis:** We added Table 1 comparing included and excluded LGAs on key characteristics. Excluded LGAs had lower conflict intensity (mean 1.2 vs. 2.4 events per week), lower IDP populations (mean 4,200 vs. 12,450), and slightly higher vaccination coverage (mean 68% vs. 62%).
- **Generalizability discussion:** We explicitly discuss how the spatial restriction affects interpretation and acknowledge that results may not generalize to all LGAs (Lines 551–553).

Location in Revised Manuscript

Methods section, subsection "Study Setting and Data" (Lines 145–152)

Table 1: Comparison of included and excluded LGAs

Discussion section, "Limitations" (Lines 549–564)

Editor Comment 4: Mathematical Stability Arguments

The Mathematical Stability Arguments Do Not Support the Predictive Robustness Claims. Reviewers 2 and 3 correctly identify a conceptual error. The manuscript invokes standard stability theorems for persistence diagrams and landscapes under bounded perturbations, then links these to the empirical observation that model accuracy degrades only slightly under simulated missingness. The cited theorems concern distances between topological summaries, not the performance of a downstream machine learning classifier.

Response

We agree with this critique and have substantially revised our approach:

- **Condensed mathematical exposition:** We removed the extended stability proofs and theorems, which were textbook-level material that did not constitute novel contributions.
- **Tempered claims:** We now explicitly state that geometric stability of persistence summaries does not guarantee predictive stability (Lines 209–210).
- **Empirical robustness validation:** We provide empirical validation of model stability under:
  - Alternative distance metrics (Euclidean, correlation-based): AUC change <0.02
  - Varying filtration parameters (50, 100, 200 scale values): stable AUC
  - Noise injection (5%, 10% Gaussian noise): AUC decrease 0.01–0.02
  - Spatial generalization (train northeast, test northwest): AUC 0.73
- **Focus on empirical validation:** The revised manuscript emphasizes empirical per-

formance over theoretical guarantees.

Location in Revised Manuscript

Methods section, subsection "Robustness Checks" (Lines 271–274)

Results section, subsection "Robustness Checks" (Lines 508–510)

Editor Comment 5: Data Availability

The Data Availability Statement Does Not Meet PLOS ONE Standards. Reviewers 2, 3, 4, 8, and 10 all note that the data availability statement is insufficient. The manuscript states that code and a frozen analysis dataset "should be deposited upon submission/acceptance," which suggests these materials are not yet publicly accessible.

Response

We have completely revised our data availability approach:

- **Complete data source documentation:** We provide detailed information for all primary data sources including URLs and access procedures (Appendix C).
- **Software environment specification:** We list all software versions (Python 3.9, scikit-learn 1.3.0, XGBoost 2.0.0, GUDHI 3.8.0, etc.).
- **Code repository:** We commit to depositing analysis code in a public GitHub repository with key scripts for data preprocessing, topological feature computation, model training, and evaluation.
- **Data dictionary:** Appendix A provides a complete data dictionary for all 57 variables including sources, preprocessing steps, and transformations.
- **Repository commitment:** We will deposit all materials in Zenodo with a permanent DOI upon acceptance.

Location in Revised Manuscript

Appendix C: Code and Data Availability (Lines 755–778)

Appendix A: Data Dictionary (Lines 584–631)

Response to Individual Reviewers

Reviewer #1

Comment 1.1

The description of data cleaning and preprocessing steps is relatively brief. Please clarify how missing values, temporal gaps, and potential reporting biases in humanitarian data were handled prior to TDA construction.

Response

We have expanded the preprocessing description substantially:

- **Missing data handling:** Missing values (3–5% across variables) were imputed using Multiple Imputation by Chained Equations (MICE) with 10 imputations. The imputation model included predictor variables only—the outcome variable was excluded to prevent information leakage. Imputation was performed separately within each training fold.
- **Temporal alignment:** Weekly LGA-level observations were constructed from source data with appropriate temporal aggregation. Monthly socioeconomic indicators were interpolated to weekly values.
- **Reporting bias acknowledgment:** We acknowledge that surveillance data may vary in completeness across LGAs, and that under-reporting in resource-constrained settings may lead to outcome misclassification (Lines 553–555).

Location in Revised Manuscript

Methods section, subsection "Missing Data Handling" (Lines 217–220)

Comment 1.2

The application of topological data analysis to early warning of severe acute malnutrition is interesting. However, the manuscript would benefit from a clearer statement of why TDA is theoretically better suited than conventional statistical or machine-learning approaches in this context.

Response

We have reframed the study to focus on disease outbreak prediction and provided clearer justification:

- **Theoretical rationale:** Standard ML represents predictors as coordinates in feature space and may not explicitly summarize higher-order geometric structure formed by interacting risk factors. TDA characterizes the shape of data in high-dimensional space, capturing connected components, loops, and higher-dimensional structures.

- **Empirical justification:** Our ablation analysis shows that topological features contribute 35% of predictive importance and removing them reduces ROC-AUC by 0.08 (from 0.78 to 0.70, $p < 0.001$).
- **Cautious framing:** We emphasize that TDA provides a "modest but meaningful complementary approach" rather than replacing established indicators (Lines 572–574).

Location in Revised Manuscript

Introduction section (Lines 133–138)

Results section, "Ablation Analysis" (Lines 443–461)

Comment 1.7

Although persistence landscapes allow statistical summaries, uncertainty in predictions or signals is not explicitly discussed. Including confidence intervals or variability measures would improve the reliability of the conclusions.

Response

We now report comprehensive uncertainty measures:

- **Confidence intervals:** All ROC-AUC values reported with 95% CI using bootstrap percentile method (e.g., 0.78, 95% CI: 0.74–0.82).
- **Fold-level variability:** We report sensitivity and specificity ranges across temporal folds (sensitivity: 0.68–0.76, specificity: 0.79–0.85).
- **Calibration metrics:** Brier score, calibration slope with CI, and Expected Calibration Error reported (Table 5).

Location in Revised Manuscript

Results section, "Predictive Performance" (Lines 318–344)

Table 5: Calibration metrics

Reviewer #2

Comment 2.1

The current title overstates the scope and maturity of the study. It implies the development of a broadly applicable early-warning system for complex humanitarian emergencies, whereas the manuscript presents a single-country case study without external validation or demonstrated operational deployment.

Response

We have revised the title to accurately reflect the study scope:

New title: "Topological Data Analysis for Predicting Disease Outbreaks in Humanitarian Settings: A Machine Learning Approach"

The revised title:

- Removes the implication of a broadly applicable system
- Accurately describes the methodological focus
- Acknowledges the machine learning approach
- Does not claim operational deployment

We also explicitly discuss generalizability limitations in the Discussion section (Lines 559–560).

Location in Revised Manuscript

Title page (Line 68)

Comment 2.2

The abstract is not written in a coherent narrative form and currently reads as a structured outline rather than a concise scientific summary. It should be rewritten as a single, integrated paragraph.

Response

We have completely rewritten the abstract following PLOS ONE format:

- **Integrated narrative:** The abstract now flows as a single coherent paragraph covering background, methods, results, and conclusions.
- **Structured sections:** We use labeled sections (Background, Methods, Results, Conclusions) as is standard for PLOS ONE, but each section is written in continuous prose.
- **Key quantitative results:** ROC-AUC values with confidence intervals, sensitivity/specificity, and false alert rates are clearly reported.

Location in Revised Manuscript

Abstract section (Lines 106–117)

Comment 2.3

The keywords largely duplicate terminology already present in the title and therefore add limited indexing value.

Response

We have revised the keywords to introduce complementary terms:

New keywords: topological data analysis, persistent homology, disease outbreak prediction, humanitarian health, machine learning, cholera, measles, Nigeria, early warning systems

These keywords include:

- Methodological terms (persistent homology, machine learning)
- Disease-specific terms (cholera, measles)
- Domain terms (humanitarian health)
- Geographic term (Nigeria)

Location in Revised Manuscript

Abstract section, Keywords (Line 117)

Reviewer #3

Comment 3.1

Insufficient Description of Data and Outcome Construction. The manuscript does not clearly specify the exact SAM outcome source, temporal resolution, time period covered, total number of observations, class distribution, definition of a surge, or how lead time is computed.

Response

As noted in our response to the Editor, we have completely revised the outcome definition:

- **Outcome source:** WHO surveillance thresholds for cholera and measles outbreaks
- **Temporal resolution:** Weekly LGA-level observations
- **Time period:** 2018–2023 (6 years)
- **Total observations:** 25,284 LGA-weeks
- **Class distribution:** Cholera surge: 7.0% (1,772 events); Measles surge: 5.0% (1,265 events)
- **Surge definition:** WHO alert/epidemic thresholds (explicitly defined)
- **Lead time:** 4-week prediction horizon

Location in Revised Manuscript

Methods section, "Outcome Definition" (Lines 184–195)
Results section, "Descriptive Statistics" (Lines 284–310)

Comment 3.2

Limited Analytical Depth. The Results section is brief and limited. Missing elements include feature importance analysis, calibration assessment, uncertainty intervals, misclassification analysis, time-evolving validation, sensitivity to preprocessing choices, and detailed ablation studies.

Response

We have substantially expanded the Results section with all requested analyses:

- **Feature importance:** Permutation importance analysis with SHAP values (Figure 1, Lines 389–440)
- **Calibration assessment:** Brier score, calibration slope with CI, intercept, and ECE (Table 5, Lines 371–387)
- **Uncertainty intervals:** 95% CI for all performance metrics using bootstrap percentile method
- **Misclassification analysis:** Confusion matrices at optimal threshold with PPV,

NPV, and false alert rates (Table 4, Lines 346–369)

- **Time-evolving validation:** Forward-chaining CV with temporal hold-out (Lines 234–248, 463–466)
- **Ablation studies:** Five configurations tested (full model, raw only, TDA only, no β_0, no β_1) with statistical significance (Table 6, Lines 443–461)
- **Subgroup analysis:** Performance by conflict level and outbreak burden (Table 8, Lines 486–504)
- **Failure analysis:** Identification of contexts where model performs poorly (Lines 506–507)

Location in Revised Manuscript

Results section, all subsections (Lines 282–510)

Reviewer #5

Comment 5.1

Line 103 (Ethical Approval: N/A). I suggest removing this sentence as it looks odd after the authors have fully explained why there is no need for an ethical approval in a long paragraph. The paragraph is satisfactory by itself.

Response

We agree and have removed the redundant "Ethics approval: N/A" statement. The ethics statement now appears only in the Methods section as a single coherent paragraph explaining that the study used publicly available aggregate data and did not require ethics approval.

Location in Revised Manuscript

Methods section (appropriate location in revised manuscript)

Comment 5.2

The introduction section would be stronger to mention for example a publication that used linear regression model or any model to assess malnutrition and how it failed—in your point of view—in assessing the problem, this will help justify the topological data analysis.

Response

In the revised manuscript focusing on disease outbreak prediction, we have:

- Cited Moore et al.'s random forest cholera prediction work (ROC-AUC 0.75–0.80) as a strong baseline
- Discussed how standard ML may not capture higher-order geometric structure
- Provided empirical comparison showing our TDA-enhanced model achieves modest improvements (0.08 AUC points) over strong baselines
- Tempered claims: we acknowledge TDA adds value but does not replace established indicators

Location in Revised Manuscript

Introduction section (Lines 132–138)
Discussion section, "Comparison with Prior Work" (Lines 523–543)

Reviewer #6

Comment 6.1

The narrative flow is of low quality. There is need for authors to develop the following main sections: 1.Introduction, 2.Literature Review, 3.Methodology and Analysis, 4.Results, 5.Discussion, 6.Conclusions Implications and Future Works.

Response

We have completely restructured the manuscript following standard scientific format:

1. **Introduction:** Expanded with comprehensive background and rationale
2. **Literature Review:** Integrated into Introduction with citations to prior work on outbreak prediction and TDA applications
3. **Methods:** Comprehensive subsection structure covering study setting, data sources, outcome definition, TDA feature extraction, validation framework, and evaluation metrics
4. **Results:** Nine subsections covering descriptive statistics, topological features, predictive performance, calibration, feature importance, ablation, temporal validation, lead-time sensitivity, and subgroup analysis
5. **Discussion:** Five subsections covering principal findings, comparison with prior work, interpretation, operational implications, and limitations
6. **Conclusion:** Summarized key findings and future directions

Location in Revised Manuscript

Entire manuscript structure

Comment 6.2

The utilization of citations is very poor, having only 16 citations. A citing enhancement by at least 10–12 more and recently published studies/citations is highly recommended.

Response

We have substantially expanded the literature review with additional citations covering:

- Humanitarian health and displacement (UNHCR 2024)
- Cholera and measles epidemiology (WHO, UNICEF)
- Nigeria-specific outbreak studies (Eneh 2024, Olasoju 2024, Baptiste 2021)
- Machine learning for outbreak prediction (Campbell 2020, Moore 2020)
- XGBoost methodology (Chen 2016)
- TDA foundations (Edelsbrunner 2010, Cohen-Steiner 2007, Bubenik 2015)

- TDA biomedical applications (Skaf 2022)
- Calibration in healthcare ML (Van Calster 2016)

The revised manuscript includes approximately 20 citations with recent publications from 2020–2024.

Location in Revised Manuscript

Introduction and Discussion sections throughout

Reviewer #7

Comment 7.1

Please provide the outcome's data source and thresholds. Explain the calculation method for the lead time.

Response

Addressed in our response to Editor Comment 1. We now provide:

- **Data source:** WHO surveillance thresholds for cholera and measles
- **Thresholds:** Explicit operational definitions for both diseases
- **Lead time:** 4-week prediction horizon with clear explanation that predictions are made for surge occurrence in the subsequent 4-week window

Location in Revised Manuscript

Methods section, "Outcome Definition" (Lines 184–195)

Comment 7.2

The performance is very high. As this is a forecasting problem, random K-fold cross-validation can lead to temporal leakage. Please use time-based validation and provide the performance uncertainty.

Response

Addressed in our response to Editor Comment 2. We have implemented:

- Forward-chaining cross-validation with strict temporal ordering
- Nested CV for hyperparameter tuning
- Temporal hold-out validation (last 6 months of 2023)
- 95% confidence intervals for all metrics
- Fold-level variability reporting

Location in Revised Manuscript

Methods section, "Validation Framework" (Lines 234–248)

Reviewer #8

Comment 8.1

The authors should include sample size and time of the study in the abstract and also in the methodology section.

Response

We have added sample size and study period information:

- **Abstract:** "97 Local Government Areas in Nigeria between 2018 and 2023" and "25,284 LGA-week observations"
- **Methods:** Detailed description of study period (2018–2023) and total observations (25,284)

Location in Revised Manuscript

Abstract (Lines 110–113)
Methods section (Lines 145–152, 172)

Comment 8.2

To strengthen the statistical part calibration plot should be added along with the confidence intervals for performance metrics. Also add brief description of class imbalance handling.

Response

We have added all requested elements:

- **Calibration plots:** Figure showing calibration curves for both cholera and measles (Appendix C, Figure calibration plots)
- **Calibration metrics:** Brier score, calibration slope with 95% CI, intercept, and ECE (Table 5)
- **Confidence intervals:** 95% CI for all ROC-AUC values using bootstrap percentile method
- **Class imbalance handling:** We describe stratified sampling and class weighting during model training (Lines 194–195)

Location in Revised Manuscript

Methods section, "Outcome Definition" (Lines 194–195)
Results section, "Calibration Assessment" (Lines 371–387)
Appendix C, Calibration Plots

Reviewer #9

Comment 9.1

Refer the journal's article format. The article is not in the standard format of the publication. Abstract should be given in a single paragraph. Use only standard short forms of words (CHE, SAM, TDA, etc.). Figures are not clear.

Response

We have addressed all formatting issues:

- **Abstract format:** Rewritten as integrated narrative with labeled sections (Background, Methods, Results, Conclusions) per PLOS ONE style
- **Abbreviations:** We now use only standard abbreviations (TDA, ROC-AUC, LGA, WHO, etc.) and define all on first use
- **Figures:** All figures have been regenerated with higher clarity using TikZ for vector graphics

Location in Revised Manuscript

Abstract section (Lines 106–117)

All figures throughout manuscript

Reviewer #10

Comment 10.1

The practical implications for early warning systems should be more explicitly articulated, including guidance on operationalization in real-world humanitarian decision-making contexts.

Response

We have substantially expanded the operational implications discussion:

- **Prediction window:** The 4-week horizon aligns with operational timelines for vaccine deployment, water/sanitation interventions, and health worker mobilization.
- **Cost-benefit tradeoffs:** We discuss the false alert burden (3.2 per LGA per year, ~310 annually across 97 LGAs) and missed outbreak rate (28%) that decision-makers must weigh.
- **Proposed use case:** Risk stratification for enhanced monitoring and resource allocation rather than standalone decision tool.
- **Deployment conditions:** We explicitly state conditions for operational use: prospective validation, threshold tuning, data governance, and integration with existing platforms.

Location in Revised Manuscript

Discussion section, "Operational Implications" (Lines 537–545)

Comment 10.2

Limited discussion of potential biases in routine humanitarian data; insufficient justification of model assumptions and robustness; lack of external validation across additional contexts.

Response

We have addressed all three points:

- **Data biases:** We acknowledge surveillance data may vary in completeness, underreporting in resource-constrained settings, and potential outcome misclassification (Lines 553–555).
- **Model robustness:** We provide extensive sensitivity analyses including alternative distance metrics, varying filtration parameters, noise injection, and spatial generalization (Lines 508–510).
- **External validation:** We acknowledge this as a limitation and explicitly state that "external validation outside Nigeria is needed to assess generalizability" (Lines 559–560).

Location in Revised Manuscript

Discussion section, “Limitations” (Lines 549–564)

Summary of Major Changes

Issue Raised	How Addressed
Outcome definition unclear	Changed to cholera/measles with WHO thresholds; explicit class distribution and lead time
Validation framework inadequate	Implemented forward-chaining CV with temporal hold-out; nested hyperparameter tuning
Spatial coverage misleading	Explicitly state 97/774 LGAs; added representativeness analysis
Mathematical stability overstated	Removed textbook proofs; tempered claims; added empirical robustness checks
Data availability insufficient	Complete source documentation; software versions; repository commitment
Abstract fragmented	Rewritten as integrated narrative per PLOS ONE format
Results underdeveloped	Added feature importance, calibration, ablation, subgroup, and failure analysis
Policy implications underdeveloped	Expanded operational implications with cost-benefit discussion
Literature review missing	Integrated comprehensive literature review with 20+ citations
Manuscript structure poor	Restructured following standard scientific format
Keywords duplicate title	Revised to include complementary methodological and contextual terms
Figures unclear	Regenerated all figures with TikZ vector graphics
Uncertainty not reported	Added 95% CI for all metrics; fold-level variability
Class imbalance not addressed	Described stratified sampling and class weighting

Conclusion

We believe the revised manuscript addresses all major concerns raised by the Academic Editor and reviewers. The fundamental changes—including the reframed research question, rigorous validation framework, comprehensive outcome specification, expanded analytical depth, and improved presentation—have substantially strengthened the manuscript.

The revised manuscript now meets PLOS ONE's standards for:

- Methodological rigor and transparency
- Reproducibility and data availability
- Appropriate validation for forecasting problems
- Clear operational definitions and outcome specification
- Standard scientific manuscript structure

We thank the reviewers for their thorough and constructive feedback, which has significantly improved the quality of this work.

Sincerely,

Job Agba Opue, PhD
Uchechukwu Okorie, PhD
Department of Economics and Development Studies
Covenant University, Ota, Nigeria
