# Supplementary material for: Topological data analysis for predicting disease outbreaks in humanitarian settings: A machine learning approach
Source: PLoS One. 2026 Jun 5;21(6):e0350644. doi: 10.1371/journal.pone.0350644 (PMC13240865; doi:10.1371/journal.pone.0350644)
Supplement: Attachment — Submitted filename: Response_to_Reviewers_auresp_3.pdf [file pone.0350644.s013.pdf]

Response to Reviewers

Manuscript PONE-D-26-03649R2

Topological Data Analysis for Predicting Disease Outbreaks in Humanitarian Settings:
A Machine Learning Approach

Dear Prof. Raimi and Editorial Team,

Thank you for the thorough and constructive review of our revised manuscript. We are grateful for the careful attention to detail and the specific guidance provided. We have addressed all points raised in the editorial letter and reviewer comments. Below, we provide a point-by-point response to each concern, with references to the corresponding changes in the revised manuscript.

Major Concerns

1. Data Availability — Insufficient Commitment for PLOS ONE Standards

The manuscript previously stated that code would be deposited upon acceptance and data were "available upon reasonable request." This was not compliant with PLOS ONE's data availability policy.

Response: We fully acknowledge this non-compliance and have made comprehensive revisions to ensure full adherence to PLOS ONE standards.

Action taken:

- **Data Availability Statement revised:** The statement now reads: "All primary data sources are publicly available (see Appendix C for URLs). The compiled dataset and analysis code are permanently archived and publicly available via Zenodo: DOI 10.5281/zenodo.19959801 (https://doi.org/10.5281/zenodo.19959801). A complete synthetic data generator reproducing the 57-variable structure is included in the replication package to enable full methodological reproduction where raw data redistribution is restricted by third-party terms."
- **Zenodo DOI provided:** We have deposited all analysis code and a frozen, de-identified dataset at Zenodo DOI 10.5281/zenodo.19959801.

- **Exact source URLs listed:** Appendix C now provides complete URLs for all primary data sources (CHIRPS, ERA5, ACLED, IOM DTM, NCDC, DHS, LSMS).
- **Synthetic data generator:** Because third-party provider terms prohibit redistribution of the compiled weekly panel, the replication package includes a complete synthetic data generator that reproduces the 57-variable structure and statistical properties, enabling full methodological reproduction.
- Location: Methods (Software and Reproducibility) and Appendix C.

2. Spatial Sampling Bias — Understated in Limitations

The reviewer noted that included LGAs differ systematically from excluded LGAs and that the manuscript did not adequately quantify how this bias might affect model generalizability.

Response: We agree that this limitation was understated and have strengthened the discussion of spatial sampling bias.

Action taken:

- Added explicit sentence in Limitations: "Because included LGAs represent higher-risk settings with elevated conflict and displacement, model performance may be lower in LGAs with lower baseline risk. Prospective validation in a representative sample of LGAs is needed before operational deployment."
- The representativeness analysis (Table 1) already quantifies the differences (conflict: 2.4 vs. 1.2 events/week; IDP: 12,450 vs. 4,200; vaccination: 62% vs. 68%).
- Location: Discussion, Limitations subsection.

3. Topological Feature Interpretation — Remains Overstated

The reviewer identified that the statement "The β_0 fragmentation index captures the degree to which risk factors operate in disconnected clusters rather than as an integrated system" represents a conceptual interpretation rather than a mathematical property, and that this interpretation has not been empirically validated.

Response: We accept this correction and have rephrased the interpretation to be more precise and cautious.

Action taken:

- Rephrased in Methods (Topological Feature Extraction): "The β_0 index mathematically counts the number of connected components in the persistence filtration. We label this

'fragmentation' as a conceptual heuristic, but this interpretation has not been empirically validated."

- The Discussion already contained appropriate cautionary language, which we have retained.
- Location: Methods, Topological Feature Extraction subsection.

4. Missing Information on Persistent Homology Computation

The reviewer noted that several critical details were missing: (1) feature scaling method, (2) justification for ϵ range, (3) whether all 57 features were used, and (4) GUDHI version and function calls.

Response: We appreciate this important methodological feedback and have added a comprehensive paragraph specifying all computational details.

Action taken:

- **Feature scaling:** Added: "Before computing Euclidean distances for the Vietoris–Rips filtration, all features were standardized to mean 0 and standard deviation 1 (z-score normalization). This scaling is essential because TDA is sensitive to the relative magnitudes of feature values."
- **ϵ range justification:** Added: "The ϵ range was chosen based on the distribution of maximum pairwise distances in the training data: the lower bound (0.01) captures local structure while the upper bound (10.0) approximates the 95th percentile of pairwise distances after standardization, ensuring that the filtration spans the relevant geometric scales without excessive computation at uninformative large scales."
- **All 57 features used:** Added explicit statement: "All 57 features were used simultaneously to compute persistence."
- **Software details:** Added: "Persistent homology computation was performed using GUDHI 3.8.0. The Vietoris–Rips complex was constructed using `gudhi.RipsComplex(points=X, max_edge_length=10.0)` followed by `create_simplex_tree()` and `persistence()` to extract persistence diagrams. Persistence landscapes were computed using `gudhi.PersistenceLandscapes` with default parameters."
- Added GUDHI citation to references.
- Location: Methods, Topological Feature Extraction subsection.

Minor Revisions

Abstract

Action taken:

- Background: Added “and” before “inadequate water and sanitation” for readability.
- Methods: Changed “surge events” to “surge events (binary indicators per LGA-week)” for clarity.
- Results: Added that validation was “temporally ordered hold-out validation” and specified that the optimal threshold was “determined using Youden’s index on validation data.”

Introduction

Action taken:

- Line 60: Corrected “Infection disease” to “Infectious disease.”

Methods

Action taken:

- Study setting: No aquaculture references found; manuscript correctly describes Nigeria LGAs.
- Data sources: Added note about expected vs. actual observations: “The expected number of observations was 97 LGAs $\times$ 52 weeks $\times$ 6 years = 30,264; the actual 25,284 represents approximately 16% missing data due to incomplete surveillance reporting in some LGA-weeks.”
- Class imbalance: Specified “class_weight=’balanced’ in XGBoost.”
- Missing data: Confirmed imputation model included all predictors but not the outcome, and that imputation was performed separately per fold with results pooled using Rubin’s rules.
- Validation framework: Added explicit statement: “Hyperparameter tuning used only the training fold data and was not permitted to access validation or test data.” Also clarified “inner 3-fold CV using only the training data.”

Results

Action taken:

- Table 2 (descriptive statistics): Added footnote for health facility density units: "Facilities per 100,000 population."
- Table 3 (predictive performance): Added footnote clarification: "determined on validation data to avoid overfitting."
- Table 6 (ablation): Added note that "P-values are from DeLong's test comparing ROC-AUCs."
- Table 7 (lead-time): Added note that "False alert rates are annualized per LGA."
- Table 8 (subgroup): Clarified that the high-conflict threshold (>10 events/week) represents "mean conflict intensity across the study period for each LGA, averaged over all weeks."

Discussion

Action taken:

- Comparison with prior work: Added sentence: "Clinical and operational significance depends on the cost of false alerts versus missed outbreaks, which is not assessed here but discussed in the operational implications section below."
- Limitations: Added new limitation: "The prediction horizon (4 weeks) was chosen post-hoc; performance at other horizons is reported but the primary horizon was not pre-specified, which increases the risk of overfitting to this horizon."

Conclusion

Action taken:

- Added: "However, routine deployment requires prospective validation and context-specific threshold tuning."

Appendices

Action taken:

- Appendix A (Data Dictionary): Clarified "Weeks since last outbreak" as "computed separately for cholera and measles."
- Appendix B (Hyperparameters): Added note that hyperparameters "were optimized via Bayesian optimization with 50 iterations, and the same search space was used for both XGBoost-Raw and XGBoost-TDA."
- Appendix C (Data Availability): Replaced placeholder GitHub URL with actual Zenodo

DOI. Removed “available upon reasonable request” language. Added explicit data availability statement compliant with PLOS ONE policy.

Language and Presentation

Action taken:

- “Infection disease” corrected to “Infectious disease” (Introduction, line 1).
- “XGBoost” and “XGBoost-Raw” consistent throughout.
- $n = 25{,}284$ properly italicized and formatted.
- Removed response-to-reviewers content from manuscript file (now provided as separate document).

Statistical and Reporting Issues

Multiple Testing

The reviewer noted that no adjustment for multiple testing was mentioned, despite many comparisons across models, outcomes, subgroups, and lead times.

Response: We have addressed this explicitly.

Action taken:

- Added new subsection “Statistical Analysis” in Methods with statement: “No adjustment for multiple comparisons was applied because the analyses are exploratory and hypothesis-generating. P-values should be interpreted descriptively. Confidence intervals and robustness checks are emphasized over formal hypothesis testing.”

Sample Size Justification

No power calculation was provided in the original submission.

Response: We have added an explicit sample size justification.

Action taken:

- Added to Methods (Statistical Analysis): “With 1,772 cholera surge events and 25,284 total observations, the study is adequately powered to detect moderate AUC differences (expected AUC ∼0.75, target AUC ∼0.80 with α=0.05, power=0.80). The effective sample size for positive events exceeds the minimum recommended for stable AUC estimation in rare-event prediction.”

Ethical Statement

The reviewer noted that the ethics statement appeared after the abstract and before the introduction, and recommended moving it to the Methods section.

Response: We have relocated the ethics statement.

Action taken:

- Ethics Statement is now positioned as subsection 2.2 within the Methods section, following Study Setting and Data (subsection 2.1).

Additional Changes

Author Addition

Action taken:

- Added Victor Ede Itita as the third author, with affiliation: Department of Social Work, University of Calabar, Calabar, Nigeria.

We believe these revisions comprehensively address all editorial and reviewer concerns. The manuscript now complies with PLOS ONE data availability requirements, provides complete methodological details for TDA computation, appropriately tempers interpretive claims, and includes all requested statistical reporting elements.

Thank you for considering our revised manuscript for publication in PLOS ONE.

Sincerely,

Job Agba Opue, PhD (Corresponding Author)
Department of Economics and Development Studies
Covenant University, Ota, Nigeria
Email: job.opue@covenantuniversity.edu.ng

On behalf of all co-authors:

- Uchechukwu Okorie, Department of Economics and Development Studies, Covenant University
- Victor Ede Itita, Department of Social Work, University of Calabar
