# Supplementary material for: Topological data analysis for predicting disease outbreaks in humanitarian settings: A machine learning approach
Source: PLoS One. 2026 Jun 5;21(6):e0350644. doi: 10.1371/journal.pone.0350644 (PMC13240865; doi:10.1371/journal.pone.0350644)
Supplement: Attachment — Submitted filename: Reviewer_Report_Reject_and_Resubmit.docx [file pone.0350644.s008.docx]

**Reviewer Report**

**Recommendation: Reject and Resubmit (Resubmission Encouraged After Substantial Revision)**

**Summary**

This manuscript proposes the use of topological data analysis (TDA), specifically persistent homology, to enhance early warning of severe acute malnutrition (SAM) in complex humanitarian emergencies in Nigeria. The conceptual integration of structural topology into humanitarian forecasting is innovative and potentially valuable. However, the current manuscript lacks sufficient empirical detail, methodological transparency, and analytical depth to support its strong performance claims.

While the conceptual integration of topological data analysis into humanitarian early warning is innovative, the manuscript lacks sufficient empirical detail, methodological transparency, and analytical depth to support its strong performance claims. Key aspects of data construction, validation design, and robustness evaluation are insufficiently documented. The manuscript currently reads more like a proof-of-concept note than a fully developed research article.

For these reasons, I recommend rejection in its current form, with encouragement to resubmit after substantial revision and expansion.

**Major Concerns**

**1. Insufficient Description of Data and Outcome Construction**

The manuscript does not clearly specify the exact SAM outcome source, temporal resolution, time period covered, total number of observations, class distribution, definition of a surge, or how lead time is computed. These omissions prevent reproducibility and limit evaluation of the reported performance.

**2. Validation Design and Risk of Information Leakage**

The reported performance (ROC–AUC 0.973; F1 0.931) is exceptionally high for humanitarian forecasting. However, the manuscript does not clearly describe whether validation is spatial, temporal, or random; whether splits prevent temporal leakage; whether TDA features are computed within each training fold; or whether hyperparameter tuning is nested within cross-validation. Without strict out-of-sample validation design, performance claims are not sufficiently supported.

**3. Limited Analytical Depth**

The Results section is brief and limited. Missing elements include feature importance analysis, calibration assessment, uncertainty intervals, misclassification analysis, time-evolving validation, sensitivity to preprocessing choices, and detailed ablation studies. For a high-dimensional modeling framework with strong predictive claims, the empirical analysis is underdeveloped.

**4. Imbalance Between Mathematical Theory and Empirical Evidence**

A substantial section is devoted to textbook-level stability theorems that are already well established in the literature. Meanwhile, empirical methodological details are comparatively brief. For an applied research article, empirical transparency and reproducibility should take precedence.

**5. Reproducibility and Data Availability**

The manuscript states that reproducibility materials should be deposited upon submission or acceptance. Code and a frozen analysis dataset (or complete preprocessing scripts) should be publicly available at submission to meet reproducibility standards.

**Minor Issues**

• Figures contain promotional-style language that should be toned down.
• “57-dimensional tensor” appears to be a feature vector and should be described accordingly.
• Interpretation of β₀ and β₁ should be empirically validated rather than asserted.
• Cost-effectiveness claims should be clearly sourced and separated from predictive performance evaluation.

**Required for Resubmission**

If the authors wish to resubmit, the manuscript would need:
1. Full and explicit outcome definition.
2. Clear, leakage-free validation design (preferably temporal and spatial hold-out).
3. Expanded results with detailed diagnostics and uncertainty analysis.
4. Public repository with reproducibility materials.
5. Reduction or condensation of textbook theoretical material.
6. More rigorous empirical justification of topological interpretation.

**Final Recommendation**

The idea is promising and innovative. However, the manuscript in its current form lacks the empirical depth, methodological transparency, and reproducibility standards required for publication. I therefore recommend rejection with encouragement to substantially revise and resubmit as a more fully developed research article.
