# Supplementary material for: Topological data analysis for predicting disease outbreaks in humanitarian settings: A machine learning approach
Source: PLoS One. 2026 Jun 5;21(6):e0350644. doi: 10.1371/journal.pone.0350644 (PMC13240865; doi:10.1371/journal.pone.0350644)
Supplement: Attachment — Submitted filename: Reviewer Comments.docx [file pone.0350644.s009.docx]

**Reviewer Comments**

The manuscript presents a relevant and interesting idea; however, several aspects require significant improvement before it can meet the publication standards of the journal.

1. **Figures**
   The figures are not sufficiently clear. It is recommended that all figures be redrawn to improve clarity, readability, and overall presentation quality.
2. **Manuscript Structure**
   Although the core concept of the study is good, the manuscript is poorly structured at present. The organization of content needs substantial revision to align with the standard scientific format expected for publication.
3. **Use of Abbreviations**
   Abbreviations should be used only for widely accepted standard terms. Currently, short forms are used excessively, including for non-standard words, which affects readability.
4. **Abstract**
   The abstract should be presented as a single, well-structured paragraph instead of multiple sections or fragmented content.

Overall, the manuscript has potential, but requires **major revisions** in presentation, structure, and clarity to reach the required publication standard.
